# Structural Characterisation of *Ophiopogon japonicus* Polysaccharides with Hypoglycaemic Activity

**DOI:** 10.3390/molecules31152659

**Published:** 2026-07-30

**Authors:** Jiani Li, Yimiao Zhou, Zhiyong Liao, Lina Deng, Xiao Liu, Zuowei Xiao

**Affiliations:** 1 Hunan Engineering and Technology Research Center for Health Products and Life Science, Hunan University of Chinese Medicine, Changsha 410208, China; 20243807@stu.hnucm.edu.cn (J.L.); lauriechow@126.com (Y.Z.);; 2 Xiangxing College, Hunan University of Chinese Medicine, Xiangyin Campus, Yueyang 414615, China; 3 Guizhou Institute of Crop Germplasm Resources, Guizhou Academy of Agricultural Sciences, Guiyang 550006, China; 4 College of Food Science and Engineering, South China University of Technology, Guangzhou 510641, China

**Keywords:** *Ophiopogon japonicus* polysaccharides, type 2 diabetes mellitus, structural characterisation, hypoglycaemic activity, zebrafish

## Abstract

Diabetes ranks first among non-communicable chronic diseases worldwide, with its incidence rising year on year; this makes the search for safer and more effective drugs an effective strategy. *Ophiopogon japonicus* (OJ) is a traditional Chinese medicinal herb used to nourish yin, and *Ophiopogon japonicus* polysaccharides (OJPS) are one of its primary active components. Currently, research into the hypoglycaemic effects of OJPS, particularly in vivo studies of its mechanisms of action, remains limited. This paper provides a systematic review of the structural characteristics of *Ophiopogon japonicus* polysaccharides and their hypoglycaemic mechanisms in a zebrafish hyperglycaemia model. Structural studies indicate that OJPS (3.929 kDa) is a highly homogeneous, highly soluble, and compact polysaccharide, primarily composed of glucose (91.85%) and galacturonic acid (3.41%). Following treatment with OJPS, hyperglycaemic zebrafish exhibited improvements in both glucose and lipid metabolism parameters. Concurrently, OJPS suppressed oxidative stress in the zebrafish, reducing hepatic lipid accumulation and inflammatory cell infiltration. Furthermore, OJPS significantly increased the expression of GLP-1, cAMP, PKA and phosphorylated CREB (P-CREB) proteins, thereby promoting insulin secretion. In summary, this study demonstrates the dual potential of OJPS in the functional food and pharmaceutical industries.

## 1. Introduction

Diabetes is a metabolic disorder characterised primarily by hyperglycaemia, accompanied by disturbances in glucose and lipid metabolism [1]. With changes in modern lifestyles and dietary habits, the incidence of diabetes has been rising year on year, affecting an increasingly younger population [2,3,4,5,6]. Diabetes affects the health of nearly 500 million people, and it is predicted that approximately 783 million people will have diabetes by 2045 [7]. However, current clinical treatments for Hyperglycaemia primarily consist of insulin and oral hypoglycaemic agents [8,9]. Long-term use of these medications is associated with various side effects, including cardiovascular [10,11], optic nerve [12,13], gastrointestinal [14,15] and hepatic complications. Numerous studies have now demonstrated that polysaccharides derived from plants possess significant hypoglycaemic activity, including those from Astragalus [16], pumpkin [17] and black fungus [18]. Owing to their minimal side effects, high safety profile and low cost, these compounds have attracted the interest of researchers both domestically and internationally.

*Ophiopogon japonicus* (OJ) is the dried tuber of *Ophiopogon japonicus* (L. f) Ker-Gawl., a plant of the Liliaceae family. It was included in the ‘Medicinal and Edible Substances’ catalogue in 2024 and possesses a wide range of pharmacological effects. *Ophiopogon japonicus* polysaccharides (OJPSs) are one of the main active components of OJ, exhibiting immunomodulatory [19], antioxidant [20] and hypoglycaemic [21] activities. Although existing studies suggest that OJPS has hypoglycaemic potential, its effects on whole-body glucose levels have not been extensively investigated, and the mechanisms by which OJPS improves hyperglycaemia remain unclear. It is therefore necessary to further elucidate the hypoglycaemic mechanisms and metabolic pathways of OJPS. Studies have shown that Ophiopogon can exert anti-inflammatory effects by activating the cAMP/PKA/CREB pathway [22]; however, no studies have reported a link between this pathway and hypoglycaemic effects.

Unlike proteins and nucleic acids, the biological activity of polysaccharides depends not only on their molecular weight, solubility and chain conformation but also on structural characteristics such as monosaccharide composition, glycosidic linkages, degree of branching and higher-order structure [21,23]. This study aims to characterise the structure of OJPS and evaluate its hypoglycaemic activity. It seeks to determine the monosaccharide composition, molecular weight distribution and glycosidic linkage patterns of OJPS. Furthermore, its hypoglycaemic effects will be assessed using a zebrafish hyperglycaemic animal model, and its molecular mechanisms will be elucidated.

## 2. Results

### 2.1. Molecular Weight Analysis of OJPS

**Figure 1:**
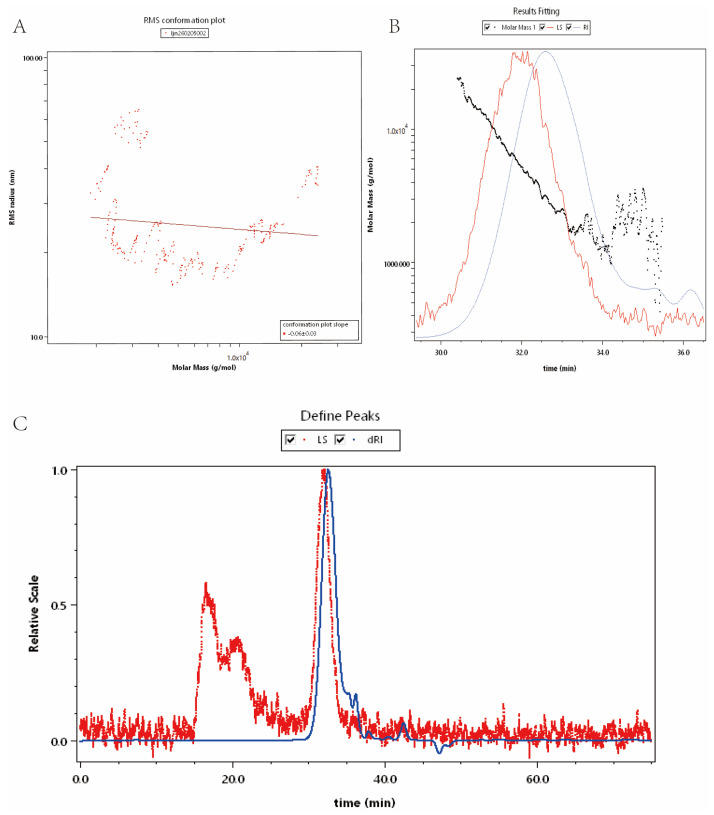
(**A**) OJPS RMS conformation plot. (**B**) OJPS absolute molecular weight analysis diagram. (**C**) OJPS molecular weight distribution chromatogram.

As shown in Figure 1B, the absolute molecular weight analysis of the OJPS, the number-average molecular weight (Mn), peak molecular weight (Mp), weight-average molecular weight (Mw), z-average molecular weight (Mz) and polydispersity index (Mw/Mn) were calculated and are presented in Table 1. The Mw of this polysaccharide is 3.929 kDa, classifying it as a typical low-molecular-weight polysaccharide, indicating high solubility. The polydispersity index is 1.472, suggesting a relatively narrow molecular weight distribution and good uniformity. The slope of the molecular conformation plot can serve as a reference for molecular conformation; the slope of −0.06 ± 0.03 shown in Figure 1A indicates that the molecular conformation of the OJPS is typically compact spherical or spheroidal, a molecular structure that is stable and not prone to rapid degradation.

### 2.2. Infrared Spectral Scan of the OJPS

**Figure 2:**
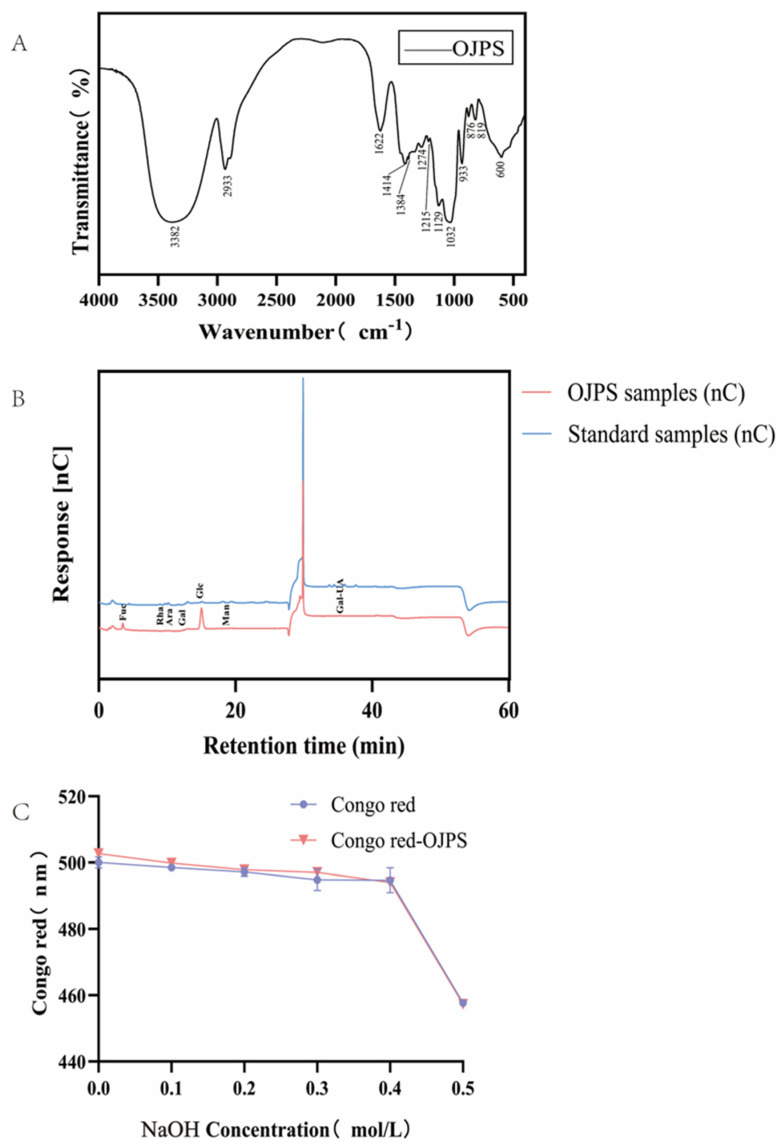
(**A**) Infrared spectrum of OJPS. (**B**) Ion chromatogram of the monosaccharide composition of OJPS. (**C**) OJPS Congo red analysis chart.

OJPS exhibits a strong, broad characteristic absorption peak at 3382.83 cm^−1^ (Figure 2A), which is attributed to the stretching vibration of the O–H group; the absorption peak at 2933.28 cm^−1^ is due to the stretching vibration of the C–H group; and the moderate characteristic absorption peak at 1622.53 cm^−1^ is attributed to the stretching vibration of the C=O group. The absorption peaks at 1414.45, 1274.43 and 1215.88 cm^−1^ originate from the stretching vibrations of the -COOH, C-OH and C=O-C groups, respectively. This indicates the presence of uronic acids and furanose rings. The FTIR spectral range of 1000–800 cm^−1^ is the fingerprint region for polysaccharide polymers; three peaks appear within this range, of which the absorption peak at 876.96 cm^−1^ is caused by the presence of α-glycosidic bonds, whilst the absorption peaks at 933.68 and 819.16 cm^−1^ are attributed to the furan ring, indicating the presence of β-glycosidic bonds. The results indicate that this OJPS sample possesses typical polysaccharide structural characteristics and contains both α-glycosidic and β-glycosidic bonds.

### 2.3. Monosaccharide Composition in OJPS

The results of the analysis of the monosaccharide composition of OJPS by ion chromatography are shown in Table 2 and Figure 2B. The results indicate that six monosaccharides and one uronic acid were detected in OJPS. Glucose is the predominant monosaccharide in OJPS, accounting for 91.85% of the total monosaccharide composition. The other components were galactose (3.41%), rhamnose (1.03%), mannose (0.97%), arabinose (0.94%), fucose (0.56%) and galacturonic acid (1.24%). These data indicate that OJPS is a glucose-dominant heteropolysaccharide. Studies have shown that polysaccharides containing glucose, galacturonic acid and mannose in their monosaccharide composition may possess good hypoglycaemic activity [24,25]. This suggests that the glucose-rich composition may be associated with hypoglycaemic activity; the OJPS samples used in this experiment may exhibit good hypoglycaemic activity, but this hypothesis requires further structure–activity relationship studies to verify.

### 2.4. Congo Red Experiment of OJPS 

The results of the Congo red assay for OJPS are shown in Figure 2C. As the concentration of NaOH increases, the trend in the maximum absorption wavelength of OJPS with Congo red is similar to that of the Congo red baseline, with no red shift observed. This indicates that OJPS lacks the rigid channel structure required to form a stable complex with Congo red and does not possess a triple-helix structure.

### 2.5. SEM Analysis of an OJPS

**Figure 3:**
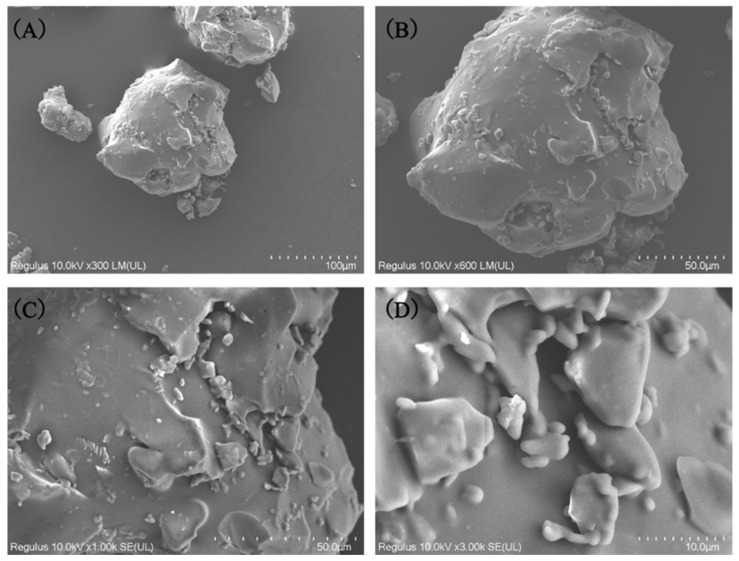
(**A**–**D**) SEM of OJPS (magnification: ×300, ×600, ×1000, ×3000).

SEM is one of the most intuitive and effective methods for observing the microstructural characteristics of polysaccharides [26]. Figure 3 shows the microstructure of OJPS at magnifications of 300, 600, 1000 and 3000 times. It can be observed that the surface of the OJPS sample is smooth and compact, with an irregular, spherical shape; the particles are relatively thick and rounded, and the surface roughness is low. Observation of the SEM image at 3000× magnification reveals that the internal structure of the OJPS sample is compact, with a flat and smooth surface and no visible network structure, indicating a high degree of purity, which is related to its molecular structure [27]. The results demonstrate that this OJPS sample possesses the typical microstructural characteristics of polysaccharides.

### 2.6. NMR Spectrum of OJPS

**Figure 4:**
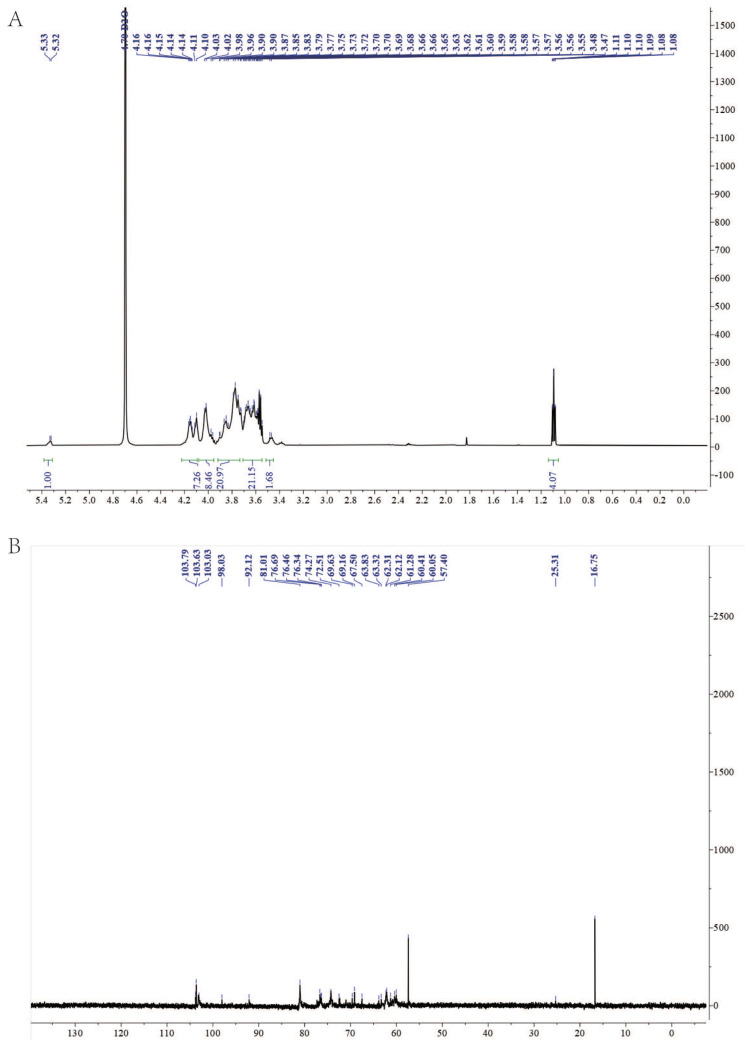
(**A**) NMR ^1^H spectrum of OJPS. (**B**) NMR ^13^C spectrum of OJPS.

The structure of OJPS was further characterised by one-dimensional NMR analysis (Figure 4). The δ value of 5.33 corresponds to a typical α-configuration anomeric hydrogen, indicating the presence of an α-furan ring in the OJPS component. No clear β-anomeric hydrogen signal was observed in the 4.3–4.5 region of the NMR spectrum; this may be due to the anomeric hydrogen of the β-glucose residues in the sample overlapping with the solvent water peak (δ ~ 4.7) or being masked by multiple peaks. The signal at δ 1.09 is a typical CH_3_ signal of a 6-deoxy sugar, indicating the presence of rhamnose (Rha) (Figure 4A). The carbon chain of the polysaccharide primarily comprises the anomeric carbon region and the ring carbon region. As shown in the figure, the three strong signals at δ 103.8, 103.6 and 103.0 correspond to C1 of β-glucose residues. Based on the chemical shifts in the anomeric carbon region of the ^13^C NMR spectrum, it is indirectly inferred that the main chain may be composed of β-glucose. However, due to the overlap of signals with the solvent peak in the ^1^H NMR spectrum, this inference awaits further verification by 2D NMR spectroscopy (Figure 4B). The above findings are consistent with the analysis of monosaccharide composition.

### 2.7. Establishment of a Hyperglycaemic Zebrafish Model

**Figure 5:**
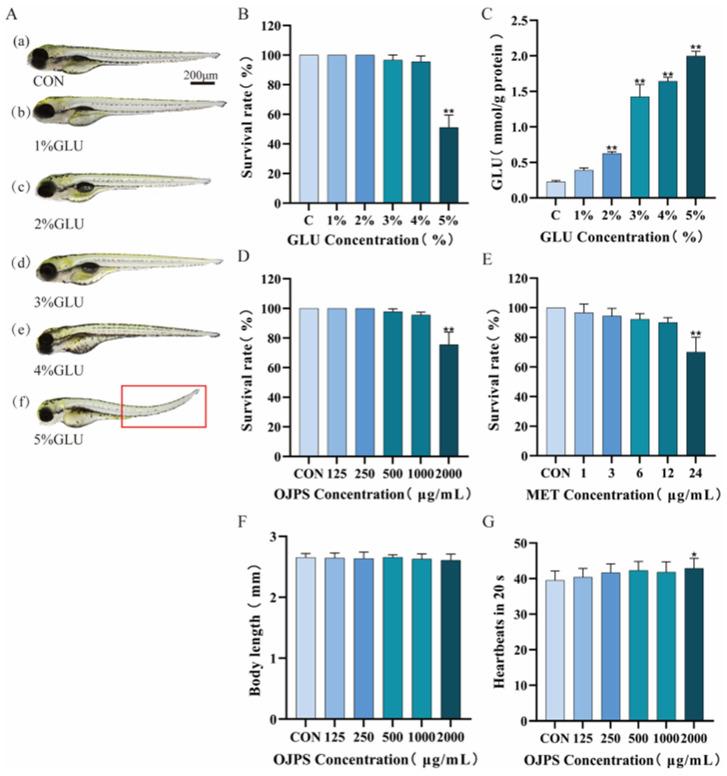
(**A**) Representative images of zebrafish larvae at various GLU concentrations (**a**–**f**). (**B**) Survival rates of zebrafish larvae at various GLU concentrations. (**C**) Whole-body glucose levels in zebrafish larvae at various GLU concentrations. (**D**) Survival rates of zebrafish larvae at various OJPS concentrations (each group *n* = 30, triplicate). (**E**) Survival rates of zebrafish larvae at various MET concentrations (each group *n* = 30, triplicate).(**F**) Body length of zebrafish larvae at various OJPS concentrations (*n* = 10).(**G**) Heart rate of zebrafish larvae at various OJPS concentrations (*n* = 10). All data were compared with the Con group, *, *p* < 0.05; **, *p* < 0.01.

A hyperglycaemic model was established by intermittently immersing zebrafish larvae in glucose solutions of varying concentrations, and GLU levels were measured in the tissues of the larvae from each group. When the whole-body glucose levels in the model group were three times higher than those in the CON group, the zebrafish model of hyperglycaemia was considered successful [28]. The results are shown in Figure 5B,C. Compared with the control group, there were significant differences in GLU levels in the zebrafish groups immersed in glucose concentrations of 2%, 3%, 4% and 5%. However, GLU levels in the larval tissues were higher than those in the control group only in the 2%, 3%, 4% and 5% glucose concentration groups. At a glucose concentration of 5%, although the GLU level was 8.86 times higher than that of the CON group, the survival rate of the zebrafish larvae decreased significantly, falling below 50%. Furthermore, microscopic examination of the zebrafish larvae revealed that some of those immersed in 5% glucose exhibited deformities such as spinal curvature, whereas no deformities were observed in the larvae from the other groups (Figure 5A). Consequently, 4% glucose was ultimately selected as the optimal concentration for inducing hyperglycaemia for subsequent experiments.

### 2.8. Acute Toxicity Tests for OJPS and Metformin

In zebrafish models, reduced hatching rates, increased heart rates and tail curvature are key parameters for assessing toxic effects. Zebrafish embryos were incubated in solutions of OJPS and MET at varying concentrations, and the effects on embryo survival rates were assessed to evaluate the toxic effects of different concentrations of OJPS and MET. A survival rate of over 80% in zebrafish embryos indicates that the compound is non-toxic and suitable for further research [29]. In the OJPS toxicity assay, compared with the control group, the survival rate of zebrafish remained above 90% at incubation concentrations ranging from 125 to 1000 μg/mL, indicating that OJPS at these concentrations had no significant effect on zebrafish survival (Figure 5D). However, at an OJPS concentration of 2000 μg/mL, embryo survival rates were significantly reduced, indicating that OJPS at this concentration inhibits zebrafish embryo development. As shown in Figure 5E, at MET concentrations of 24 μg/mL, the survival rate of zebrafish larvae decreased to below 80% after 6 days of incubation, showing a significant difference compared with the CON group (*p* < 0.01). Monitoring heart rate can be used to assess whether the sample concentration has an adverse effect on zebrafish. As shown in Figure 5F,G, a slight increase in heart rate was observed at a sample concentration of 2000 μg/mL (*p* < 0.05), but no significant inhibition of body length development was observed.

Based on the effects of different concentrations of MET and OJPS on the survival rate and development of zebrafish larvae, it was ultimately decided to select OJPS-L, OJPS-M and OJPS-H at concentrations of 600, 800 and 1000 μg/mL, respectively, and MET at a concentration of 12 μg/mL, for use in subsequent further studies.

### 2.9. The Effect of OJPS on Whole-Body Glucose Levels in Zebrafish Larvae with Hyperglycaemic

**Figure 6:**
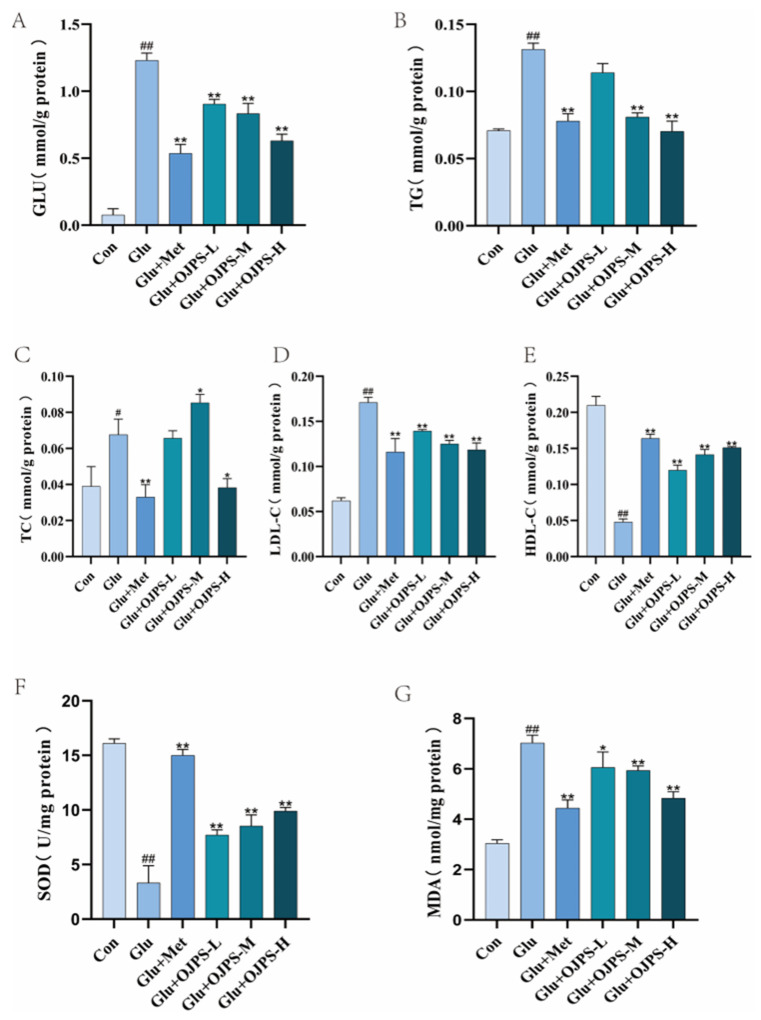
The effect of OJPS on GLU, TG, TC, LDL-C, HDL-C, SOD, and MDA levels in hyperglycaemic zebrafish. (**A**) GLU content. (**B**) TG content. (**C**) TC content. (**D**) LDL-C content. (**E**) HDL-C content. (**F**) SOD content. (**G**) MDA content. For each group, *n* = 9. #, *p* < 0.05, ##, *p* < 0.01 compared with the Con group; **, *p* < 0.01, *, *p* < 0.05 compared with the GLu group.

In previous studies, we investigated the use of glucose concentration to establish a zebrafish model of hyperglycaemia and found that intermittent stimulation with 4% glucose effectively established a hyperglycaemic zebrafish model. Therefore, in this experiment, Met and OJPS interventions were administered concurrently with high-concentration glucose stimulation, and glucose levels in the tissues of zebrafish larvae were measured following the conclusion of the interventions. When the whole-body glucose levels of the GLU group were three times higher than those of the control group, the establishment of a hyperglycaemic zebrafish model was considered successful. As shown in Figure 6A, the whole-body glucose level in the GLU group was (1.23 ± 0.053 mmol/g), approximately 15 times higher than that of the control group (0.077 ± 0.045 mmol/g) (*p* < 0.01), indicating that the hyperglycaemic zebrafish model was successfully established after 6 days of intermittent immersion in glucose solution.

At the same time, compared with the model group, the GLU+Met group and the GLU+OJPS (600, 800, 1000 μg/mL) groups both significantly reduced the glucose content in the tissues of zebrafish larvae (all *p* < 0.01), and the effect was dose-dependent. These results indicate that OJPS has a significant hypoglycaemic effect on zebrafish with type 2 diabetes, with the highest dose of OJPS (1000 μg/mL) being the most effective.

### 2.10. The Effects of OJPS on Lipid Metabolism in Zebrafish Larvae with T2DM

Obesity or being overweight is one of the key risk factors for diabetes, doubling the risk of developing the condition [30,31]. As diabetes and its complications affect many aspects of physiology, the benefits of weight loss extend beyond whole-body glucose level control and are also associated with numerous cardiovascular risk factors [32,33]. Serum TC and TG are important indicators for assessing cholesterol metabolism in the body; HDL-C is the primary transport carrier from extrahepatic tissues to the liver, whilst LDL-C is the primary transport carrier from the liver to tissues and cells throughout the body [34,35]. Consequently, TC, TG, HDL-C and LDL-C are frequently used as markers for detecting dyslipidaemia.

As shown in Figure 6B–D, compared with the CON group, the GLU group exhibited significant increases in TC (*p* < 0.05), TG (*p* < 0.01) and LDL-C (*p* < 0.01), presenting the typical hyperlipidaemic symptoms of a type 2 diabetes zebrafish model. Among these, compared with the GLU group, the MET group and the OJPS-L, OJPS-M, and OJPS-H groups all showed a significant downward trend in TC and TG; the OJPS-L group reduced the levels, but this was not statistically significant (*p* > 0.05). At the same time, compared with the GLU group, the MET group and the OJPS-L, OJPS-M, and OJPS-H groups all showed a significant downward trend in LDL-C (*p* < 0.01). Furthermore, as shown in Figure 6E, compared with the CON group, HDL-C in the GLU group showed a significant downward trend (*p* < 0.01). Compared with the GLU group, HDL-C in the MET group and the OJPS-L, OJPS-M, and OJPS-H groups all showed a significant upward trend (all *p* < 0.01). The OJPS-L, OJPS-M and OJPS-H groups exhibited a dose-dependent effect. This is consistent with the findings of Zhang [36], indicating that OJPS can reduce lipid levels in body tissues, alleviate lipid metabolism disorders, and thereby improve glycaemic control.

### 2.11. The Effects of OJPS on Oxidative Stress in Zebrafish Larvae with T2DM

Dysregulated lipid metabolism and inflammation both contribute to the pathogenesis of type 2 diabetes mellitus (T2DM) [37,38]. Relevant studies have shown that excessive lipid accumulation induces oxidative stress in the body, leading to an imbalance in the antioxidant defence system and further disrupting lipid metabolic pathways. Superoxide dismutase (SOD) is a metalloproteinase that scavenges superoxide anion radicals and serves as an indicator of the body’s ability to resist oxidative damage [34,35].

The effects of OJPS on SOD activity and MDA levels in hyperglycaemic zebrafish larvae are shown in Figure 6F,G. Compared with the CON group, SOD activity was significantly reduced in the GLU group (*p* < 0.01), whilst MDA levels were significantly elevated (*p* < 0.01). As SOD activity and MDA levels were negatively correlated, this indicates that glucose-soaked feeding reduces the activity of antioxidant enzymes in zebrafish larvae, leading to oxidative stress. Compared with the GLU group, SOD activity in the MET group and the OJPS-L, OJPS-M, and OJPS-H groups all showed a significant upward trend (all *p* < 0.01), whilst MDA levels in the MET group and the OJPS-L, OJPS-M, and OJPS-H groups were significantly reduced (*p* < 0.05 and *p* < 0.01, respectively). Following intervention with OJPS, antioxidant enzyme activity increased, indicating that OJPS can alleviate oxidative stress induced by high glucose concentrations in zebrafish larvae. By alleviating oxidative stress in the body, it improves the oxidative breakdown and metabolism of glucose, thereby regulating whole-body glucose levels.

### 2.12. Histopathological Analysis

**Figure 7:**
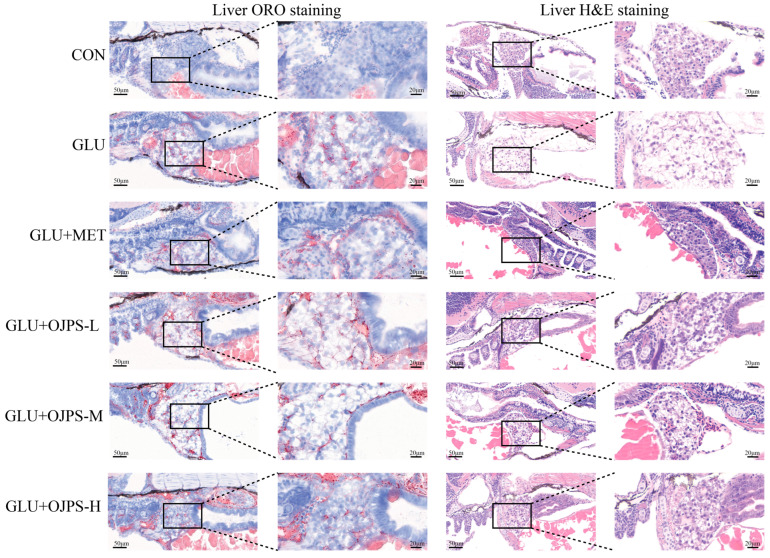
HE and Oil Red O stained liver sections from zebrafish larvae in each group.

Liver tissue sections from zebrafish larvae were stained with Haematoxylin and Eosin (HE) and Oil Red O to assess histological changes and lipid accumulation during the experiment (Figure 7). H&E staining allows for a clear assessment of hepatic steatosis and inflammatory responses, whilst Oil Red O staining facilitates further observation of lipid accumulation within the liver tissue [39,40]. Examination of the H&E and Oil Red O-stained sections revealed that the liver tissue of the CON group of juvenile fish exhibited normal morphology, with tightly packed hepatocytes; no inflammatory cell infiltration or lipid vacuoles were observed, nor was there any significant accumulation of red lipid droplets in the intercellular spaces. In contrast, the pathological sections of the GLU group showed marked hepatocyte degeneration and vacuolisation, with a distinct accumulation of red lipid droplets in the intercellular spaces. However, following MET treatment, inflammatory cell infiltration and vacuolisation were reduced to varying degrees, with hepatocytes arranged in an orderly manner and the tissue appearing well organised. Compared with the CON group, following treatment with OJPS-L, OJPS-M or OJPS-H, the lipid droplets and vacuoles within the hepatocyte interstices showed varying degrees of reduction, and the hepatocyte arrangement was more orderly, consistent with the trend observed in the MET group. The improvement exhibited a certain degree of dose dependency, with the zebrafish liver tissue morphology being optimal following treatment with OJPS-H, indicating superior therapeutic efficacy.

### 2.13. OJPS Activates the GLP-1/cAMP/PKA/CREB/INS Pathway

**Figure 8:**
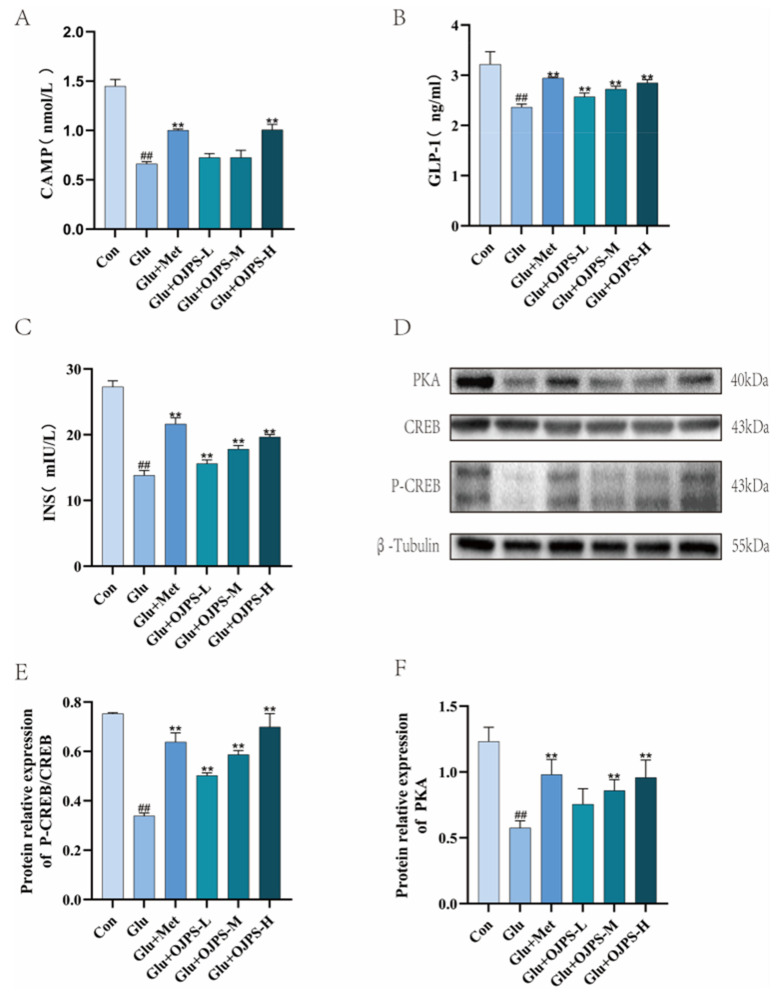
(**A**) The effect of OJPS on CAMP levels in hyperglycaemic zebrafish. (**B**) The effect of OJPS on GLP-1 levels in hyperglycaemic zebrafish. (**C**) The effect of OJPS on INS levels in hyperglycaemic zebrafish. (**D**) The relative protein quantification of PKA, CREB, P-CREB, β-Tubulin. (**E**) The relative protein quantification of CREB/P-CREB. (**F**) The relative protein quantification of PKA. For each group, *n* = 3. ##, *p* < 0.01 compared with the Con group; **, *p* < 0.01, compared with the GLu group.

The protein levels of GLP-1, cAMP, PKA, P-CREB and INS in zebrafish tissues were measured using enzyme-linked immunosorbent assay (ELISA) and Western blot analysis (Figure 8). The expression of GLP-1, cAMP, PKA, P-CREB and INS in zebrafish tissues from the GLU group was significantly lower than that in the CON group. Western blot analysis revealed that the OJPS and MET groups significantly increased PKA levels and the phosphorylation levels of CREB (*p* < 0.01) (Figure 8D). Concurrently, ELISA kit results indicated that the OJPS and MET groups significantly increased GLP-1, cAMP and INS levels (*p* < 0.01). These findings demonstrate that OJPS significantly upregulates GLP-1 levels whilst simultaneously increasing cAMP content, enhancing PKA activity and promoting CREB phosphorylation in zebrafish, ultimately leading to increased INS expression.

## 3. Materials and Methods

### 3.1. Materials and Reagents

Ophiopogon polysaccharides (S27672, 98% pure), glucose, metformin, PBS and saline were purchased from Shanghai Yuanye Co., Ltd. (Shanghai, China)Glucose (GLU, mmol/g), total triglycerides (TG, mmol/L), total cholesterol (TC, mmol/L), low-density lipoprotein cholesterol (LDL-C, mmol/L), high-density lipoprotein cholesterol (HDL-C, mmol/L), superoxide dismutase (SOD, U/mg protein), malondialdehyde (MDA, nmol/mg protein) and cyclic adenosine monophosphate (cAMP, nmol/L) were supplied by Nanjing Jiancheng Co., Ltd. (Nanjing, China) and Quanzhou Ruixin Biotechnology Co., Ltd. (Quanzhou, China).

### 3.2. Animals

Sexually mature wild-type male and female zebrafish are placed in a breeding tank fitted with a partition in a ratio of 1:1 or 1:2 and left undisturbed overnight. The following morning, the water level in the tank is reduced, the partition is opened and the lamp switched on; the adult fish will begin spawning once the light is turned on, and the embryos are collected 1–2 h later. The embryos were transferred to a petri dish; an appropriate volume of embryo culture medium was added; and the dish was placed in a constant temperature and humidity incubator at 28 ± 1 °C, with a 14-h light and 10-h dark cycle, and cultured until 24 h post-fertilisation (24 hpf). Well-developed and healthy embryos were selected for subsequent experiments. This study has been approved by the Institutional Animal Care and Use Committee (IACUC) of Hangzhou Huante Biotechnology Co., Ltd. (Hangzhou, China) (IACUC-2024-10402-01).

### 3.3. Molecular Weight Analysis of OJPS

Weigh a specific amount of OJPS and dissolve it in a 0.1 M NaNO_3_ aqueous solution (containing 0.02% NaN_3_, *w*/*w*) to achieve a final concentration of 1 mg/mL; filter the solution through a 0.45 μm filter before analysing it on the instrument. HPGPC was used to determine the molecular weight under the following conditions: a series connection of Ohpak SB-805 HQ (300 × 8 mm) and Ohpak SB-803 HQ (300 × 8 mm) gel exclusion chromatography columns; column temperature, 45 °C; injection volume, 100 μL; mobile phase A (0.02% NaN_3_, 0.1 M NaNO_3_); flow rate, 0.6 mL/min; and elution gradient, isocratic elution for 75 min.

### 3.4. Infrared Spectral Scan of the OJPS

Weigh a small amount of OJPS, mix it thoroughly with 200 mg of potassium bromide, and press the mixture into 1 mm thick discs using a mould. Using thin slices of pure potassium bromide as a blank, measure the blank background; then, using an FT-IR spectrometer, analyse the sample in the range of 4000 cm^−1^ to 400 cm^−1^ to identify its characteristic functional groups.

### 3.5. Determination of Monosaccharide Composition in OJPS

Take a clean chromatography vial, weigh out an appropriate amount of OJPS sample, add 1 mL of 2 M TFA solution, and heat at 121 °C for 2 h. Purge with nitrogen and dry. Wash with 99.99% methanol; after drying, repeat the washing with methanol 2–3 times. Dissolve in sterile water, and transfer to a chromatography vial for analysis. Use a Dionex CarboPac PA20 (150 × 3.0 mm, 10 μm) (Thermo Fisher Scientific, Waltham, MA, USA) liquid chromatography column, with a mobile phase of 0.1 M NaOH, an injection volume of 5 μL, a flow rate of 0.5 mL/min, and a column temperature of 30 °C.

### 3.6. OJPS Congo Red Experiment

Following the method described by Wang [41] with minor modifications, distilled water was used as the blank control. A 1 mg/mL OJPS solution was scanned using a UV spectrophotometer in the wavelength range of 400–600 nm. A standard curve was plotted with the final concentration of NaOH on the *x*-axis and the wavelength of maximum absorption (λ) on the *y*-axis.

### 3.7. SEM Scan of OJPS

A small amount of OJPS was placed on a metal stage, coated with a thin layer of gold using an ion sputtering system (Model SBC-12), and examined using a Regulus 8100 scanning electron microscope (Hitachi High-Tech Corporation, Tokyo, Japan) at magnifications ranging from 500 to 10,000.

### 3.8. NMR Analysis of OJPS

Weigh out approximately 5 mg of the OJPS, dissolve it in pure water, transfer it to an NMR tube, and use an NMR spectrometer to acquire and record the chemical shifts of ^1^H NMR and ^13^C NMR.

### 3.9. Establishment of a Hyperglycaemic Zebrafish Model

Zebrafish embryos were collected 24 h after fertilisation and randomly divided into six groups, each containing 30 zebrafish embryos. These were designated as the blank control group and the 1%, 2%, 3%, 4% and 5% glucose model groups. The control group (CON) was immersed in embryo culture medium, whilst the glucose model groups were immersed in 1%, 2%, 3%, 4% and 5% glucose solutions. For the glucose model groups, the glucose solution and embryo culture medium were replaced every 24 h until day 6 post-fertilisation (6 dpf). The survival rates of each group were recorded, and the development of zebrafish embryos in each group was observed under a microscope. Finally, whole-body glucose levels were measured in the tissues of zebrafish larvae from each group.

### 3.10. Acute Toxicity Tests for OJPS and MET

Zebrafish embryos 24 h post-fertilisation were selected for acute toxicity testing with OJPS and MET. The OJPS solution concentration gradients were set at 0, 125, 250, 500, 1000 and 2000 μg/mL, whilst the metformin solution concentration gradient was set at 0, 1, 3, 6, 12 and 24 μg/mL. The embryos were transferred between the different treatment concentrations and the embryo culture medium every 24 h. The solution was replaced every 24 h, and the mortality rate was recorded.

### 3.11. Experimental Procedures for Zebrafish Larvae

The hyperglycaemic zebrafish model was established using intermittent stimulation with high-concentration glucose for 6 days [42]. The results of the above experiments indicate that the optimal modelling concentration is a 4% glucose solution, and the dosing concentrations for MET and OJPS solutions have been determined. Zebrafish embryos were collected 24 h post-fertilisation and randomly divided into six groups, each comprising 30 zebrafish larvae: the CON group, the GLU group, the GLU+MET group (12 μg/mL), the GLU+OJPS-L group (600 μg/mL), the GLU+OJPS-M group (800 μg/mL), and the GLU+OJPS-H group (1000 μg/mL). The groups were reared in 6-well plates under standard laboratory rearing conditions. The blank control group received no treatment; for the other groups, the respective solutions were alternated with the embryo culture medium every 24 h until day 6, at which point the zebrafish larvae were examined following the conclusion of the modelling and OJPS interventions.

### 3.12. Histopathological Analysis

Following the completion of drug administration, six zebrafish larvae were selected from each group and fixed in a 4% paraformaldehyde solution. The experiment was repeated independently three times. In each repetition, three of the six fixed juveniles were randomly selected for Oil Red O staining, whilst the remaining three were subjected to Haematoxylin and Eosin (HE) staining. Pathological changes in the zebrafish liver tissue were observed under a light microscope.

### 3.13. Biochemical Analysis

Thirty 6-day-post-fertilisation (6 dpf) zebrafish larvae from each group, following incubation in high-concentration glucose and OJPS, were mechanically homogenised in PBS at a weight-to-volume ratio of 1:9 (g/mL). The samples were centrifuged (6000 r/min, 15 min), and the supernatant was collected to measure biochemical parameters in the tissue homogenate, including glucose content (GLU, mmol/g), total triglycerides (TG, mmol/L), total cholesterol (TC, mmol/L), low-density lipoprotein cholesterol (LDL-C, mmol/L), high-density lipoprotein cholesterol (HDL-C, mmol/L), superoxide dismutase (SOD, U/mg protein), malondialdehyde (MDA, nmol/mg protein) and cyclic adenosine monophosphate (cAMP, nmol/L). The assay kits were supplied by Nanjing Jiancheng Co., Ltd. and Quanzhou Ruixin Biotechnology Co., Ltd. All assay kits were subjected to rigorous inspection and analysis in accordance with the standards and specifications established by the manufacturers.

### 3.14. Western Blotting Analysis

The expression levels of the target protein were compared across the various groups of zebrafish larvae to evaluate the efficacy of OJPS treatment. Collect 90 zebrafish larvae from each group (with no fewer than 30 larvae per group), and wash them twice with pre-chilled PBS buffer. Add RIPA lysis buffer containing protease inhibitors and phosphatase inhibitors at a weight-to-volume ratio of 1:9 (g/mL), and homogenise the zebrafish tissue using a tissue homogeniser. Total proteins were extracted from zebrafish tissues using a protein lysis buffer. Following lysis on ice for 1 h, the samples were centrifuged at 6000 rpm for 15 min. The supernatant was collected and the protein concentration was determined using the BCA method. After adjusting the proteins to the same concentration, they were loaded onto a 10% SDS-PAGE gel and then transferred to a polyvinylidene fluoride membrane. Following membrane transfer, block for 1 h in 5% skimmed milk, then incubate at 4 °C with primary antibodies against PKA, CREB, P-CREB and β-tubulin, followed by incubation with secondary antibodies at room temperature for 1 h. Visualise using a chemiluminescence imaging system.

### 3.15. Statistical Analysis

Experimental data are presented as mean ± standard deviation (X ± SD). Analysis of variance (ANOVA) was performed using GraphPad Prism 10 software, and *t*-tests were used to assess statistical significance, with *p* < 0.05 and *p* < 0.01 taken as indicators of statistical significance. GraphPad Prism 10 and Adobe Illustrator 2026 were used to generate and process graphical data.

## 4. Discussion

Chronic hyperglycaemia is caused by various environmental factors. GLP-1 is an incretin hormone secreted by intestinal L-cells in response to nutritional intake; it promotes insulin secretion, effectively regulates postprandial whole-body glucose levels, and plays a key role in glucose metabolism [43,44]. Based on this mechanism, GLP-1 receptor agonists have been developed as novel antihyperglycaemic drugs for the treatment of T2DM [45]. Currently, research into polysaccharides extracted from natural products that exert hypoglycaemic effects by stimulating GLP-1 secretion is attracting widespread attention. Previous studies have shown that polysaccharides from white lupin [46], bergamot [47] and Dendrobium officinale [48] can promote GLP-1 secretion by activating upstream signalling pathways.

However, our understanding of the complete signalling pathway by which polysaccharides systematically activate GLP-1, ultimately promoting insulin secretion, remains limited. Against this background, this study selected OJPS as the subject of investigation. By characterising its structure and evaluating its hypoglycaemic activity using zebrafish as a model, the study aimed to elucidate the structure–activity relationship between the structural features of the polysaccharide and its hypoglycaemic activity. The results indicate that OJPS treatment is associated with the activation of the GLP-1/cAMP/PKA/CREB pathway and elevated insulin levels, suggesting that this pathway may be involved in the hypoglycaemic action of OJPS. This provides a theoretical basis for the development of natural hypoglycaemic agents.

Research indicates that OJPS is a polysaccharide composed primarily of glucose as its monosaccharide component, featuring a smooth surface and a compact, spherical-like internal structure. Furthermore, with a molecular weight (Mw) of 3.929 kDa, it is a low-molecular-weight polysaccharide with high solubility. Although metformin (a small-molecule drug) and OJPS (a high-molecular-weight polysaccharide) were used at markedly different concentrations, both doses were validated as safe through preliminary toxicity assays. As a high-throughput in vivo model, the zebrafish offers advantages such as low cost, short experimental cycles and ease of observation. Consequently, this study utilised zebrafish as the experimental animals and established a hyperglycaemic model by intermittently immersing them in a 4% glucose solution. The results from the zebrafish experiments indicate that OJPS reduces whole-body glucose levels in zebrafish, accompanied by increased insulin levels, and alleviates lipid metabolism disorders and oxidative stress. Histopathological analysis shows that OJPS significantly improves hepatic steatosis and inflammatory responses and reduces lipid accumulation in the liver. ELISA and Western blot results indicate that OJPS significantly increases PKA protein expression and promotes CREB protein phosphorylation in zebrafish. These findings suggest that the hypoglycaemic effect of OJPS may be related to the regulation of the GLP-1/cAMP/PKA/CREB signalling pathway; however, a causal relationship requires further validation through inhibitor experiments.

However, this study does have certain limitations. Firstly, with regard to the structural characterisation of Ophiopogon polysaccharides, no further investigations were conducted, such as methylation analysis or two-dimensional NMR. Secondly, regarding the mechanism of action, there is a lack of further validation using GLP antagonists to confirm that OJPS improves hyperglycaemia in zebrafish by modulating the GLP signalling pathway. Furthermore, as this study utilised only zebrafish as experimental animals, further research in mammalian models is required. In future studies, these limitations will be addressed through more in-depth investigation.

## 5. Conclusions

This study indicates that OJPS is a heteropolysaccharide composed primarily of glucose, which accounts for 91.85 per cent of its composition. First, zebrafish toxicity experiments were conducted. By observing and recording the survival rates and growth and development of zebrafish larvae, the concentrations required to induce hyperglycaemia, as well as those for OJPS and MET administration, were determined. The results of the zebrafish experiments showed that OJPS significantly reduced glucose levels in larval tissues; increased levels of TC, TG and LDL-C; and reduced HDL-C levels. This indicates that OJPS can effectively improve glucose and lipid metabolism disorders in zebrafish larvae and effectively alleviate symptoms of hyperglycaemia. Furthermore, OJPS significantly reduced MDA levels and significantly increased SOD activity, suggesting that it can alleviate oxidative stress in the larvae. Histopathological analysis revealed that OJPS improved liver tissue lesions and reduced lipid droplet accumulation in zebrafish larvae. WB results showed that OJPS significantly increased the expression of PKA and P-CREB proteins in the fry’s tissues. Furthermore, ELISA results indicated that OJPS significantly upregulated the levels of GLP-1, cAMP and INS. The study suggests that the hypoglycaemic effect of OJPS may be related to the regulation of the GLP-1/cAMP/PKA/CREB signalling pathway. This study lays a scientific foundation for understanding the structure–activity relationship of polysaccharides; as a natural hypoglycaemic agent, OJPS holds immense potential for further research and development.

## Figures and Tables

**Table 1:** Molecular weight of OJPS.

Name	Molecular Weight
(Mn)	2.670 kDa
(Mp)	3.180 kDa
(Mw)	3.929 kDa
(Mz)	6.337 kDa
Polydispersity (Mw/Mn)	1.472

**Table 2:** Monosaccharide composition and content of OJPS.

Index Monosaccharide Molar Ratio	Value
Glucose (%)	91.85
Galactose (%)	3.41
Galacturonic acid (%)	1.24
Rhamnose (%)	1.03
Mannose (%)	0.97
Arabinose (%)	0.94
Fucose (%)	0.56

## Data Availability

The original contributions presented in this study are included in the article. Further inquiries can be directed to the corresponding authors.
